# Type and timing of childhood maltreatment and reduced visual cortex volume in children and adolescents with reactive attachment disorder

**DOI:** 10.1016/j.nicl.2018.07.018

**Published:** 2018-07-23

**Authors:** Takashi X. Fujisawa, Koji Shimada, Shinichiro Takiguchi, Sakae Mizushima, Hirotaka Kosaka, Martin H. Teicher, Akemi Tomoda

**Affiliations:** a Research Center for Child Mental Development, University of Fukui, 23-3 Matsuoka-Shimoaizuki, Eiheiji-cho, Fukui 910-1193, Japan; b Division of Developmental Higher Brain Functions, United Graduate School of Child Development, University of Fukui, 23-3 Matsuoka-Shimoaizuki, Eiheiji-cho, Fukui 910-1193, Japan; c Biomedical Imaging Research Center, University of Fukui, 23-3 Matsuoka-Shimoaizuki, Eiheiji-cho, Fukui 910-1193, Japan; d Department of Child and Adolescent Psychological Medicine, University of Fukui Hospital, University of Fukui, 23-3 Matsuoka-Shimoaizuki, Eiheiji-cho, Fukui 910-1193, Japan; e Department of Neuropsychiatry, University of Fukui, 23-3 Matsuoka-Shimoaizuki, Eiheiji-cho, Fukui 910-1193, Japan; f Department of Psychiatry, Harvard Medical School, Boston, MA, USA; g Developmental Biopsychiatry Research Program, McLean Hospital, Belmont, MA, USA

**Keywords:** Childhood maltreatment, Reactive attachment disorder (RAD), Voxel-based morphometry, Gray matter (GM) volume, Visual cortex, Sensitive period

## Abstract

Reactive attachment disorder (RAD) is a severe social functioning disorder associated with early childhood maltreatment where the child displays emotionally withdrawn/inhibited behaviors toward caregivers. Brain regions develop at different rates and regions undergoing rapid change may be particularly vulnerable during these times to stressors or adverse experiences. The aim of this study was to investigate the effect of type and timing of childhood adversities on structural alterations in regional gray matter (GM) volume in maltreated children with RAD.

High-resolution magnetic resonance imaging datasets were obtained for children and adolescents with RAD (*n* = 21; mean age = 12.76 years) and typically developing (TD) control subjects (*n* = 22; mean age = 12.95 years). Structural images were analyzed using a whole-brain voxel-based morphometry approach and the type and timing of maltreatment, which may be more strongly associated with structural alterations, was assessed using random forest regression with conditional inference trees.

Our findings revealed that there is a potential sensitive period between 5 and 7 years of age for GM volume reduction of the left primary visual cortex (BA17) due to maltreatment. We also found that the number of types of maltreatment had the most significant effect on GM volume reduction and that the second most significant variable was exposure to neglect.

The present study provides the first evidence showing that type and timing of maltreatment have an important role in inducing structural abnormalities in children and adolescents with RAD.

## Highlights

• We studied the effect of maltreatment type and timing on brain structure in RAD.
• There was a sensitive period for GM volume reduction in the visual cortex.
• The clinical symptoms of RAD showed the same sensitive period.
• The number of types of maltreatment had significant effects on GM volume reduction.
• Neglect was common and could be acting as a basis for the effects of maltreatment.

## 1 Introduction

Reactive attachment disorder (RAD) is a severe social functioning disorder associated with early childhood maltreatment where the child displays emotionally withdrawn/inhibited behaviors toward caregivers according to the 5th edition of the Diagnostic and Statistical Manual of Mental Disorders (DSM-5) (American Psychiatric Association, 2013). The prevalence of RAD based on the DSM-IV criteria, in which RAD (the inhibited type of RAD) and disinhibited social engagement disorder (the disinhibited type of RAD) are not completely independent, is reportedly 19.4–40.0% among maltreated children in foster care (Lehmann et al., 2013; Zeanah et al., 2004) and 1.4–2.4% among children in the general population (Minnis et al., 2013). Children with RAD are more likely to have multiple comorbidities, such as with attention deficit-hyperactivity disorder (52%), post-traumatic stress disorder (PTSD; 19%), and autism spectrum disorder (14%) (Minnis et al., 2013; Pritchett et al., 2013). Thus, although RAD has high prevalence and presents with various difficulties on pathological assessment, there have been very few investigations into the possible neurobiological consequences of RAD.

The childhood brain is considered to have specific temporal sensitivity for each brain region in terms of both structure and function (Andersen and Teicher, 2008; Andersen et al., 2008). Adverse experiences during these sensitive periods may affect brain organization, which is under rapid development, such as synaptic production, pruning, and myelination (Teicher et al., 2002). From the perspective of neurobiology, the brain during childhood is highly plastic and develops by constant modification based on gene-environment interactions (Teicher and Samson, 2013). The developmental curves of cortical gray matter volume are regionally specific and have distinctive trajectories for each local region (Giedd et al., 1999; Gogtay et al., 2004). Therefore, the reactivity of the brain to the developmental environment may also differ depending on the developmental stage. Similarly, a number of previous studies has shown that different types of adversity exert effects on different brain areas (Choi et al., 2009; Choi et al., 2012; Heim et al., 2013; Tomoda et al., 2011; Tomoda et al., 2012). Thus, it also seems that the clinical phenotypes of maltreatment during childhood may partially depend on the type or timing of exposure to adversities (Khan et al., 2015; Schalinski and Teicher, 2015; Teicher et al., 2018).

The aim of this study was to investigate the effect of type and timing of childhood adversities on structural alterations in regional gray matter (GM) volume in maltreated children with RAD. In our previous study, we found that children and adolescents with RAD exhibit structural abnormalities in the left primary visual cortex (BA17) (Shimada et al., 2015). Similar to the trends found in related studies (Andersen and Teicher, 2008; Andersen et al., 2008; Tomoda et al., 2011; 2012), we hypothesized that structural abnormalities in RAD would be dependent on the type or timing of the maltreatment. Hence, we sought to determine if there were windows of vulnerability across age and type of maltreatment for structural alteration in children and adolescents with RAD.

## 2 Methods

Subject information, such as demographic and clinical characteristics of RAD and typically developing (TD) groups, the acquisition condition, and processing of the structural imaging data have been described in our previous study (Shimada et al., 2015) where further details can be found.

### 2.1 Participants

Twenty-one medication-naive 10- to 17-year-old children with a clinical diagnosis of RAD participated in the study (mean age = 12.8 years; 13 girls), as previously reported (Shimada et al., 2015). Participants were right handed and their race/ethnicity was Japanese. Participants were recruited from the Department of Child and Adolescent Psychological Medicine at the University of Fukui Hospital. RAD was assessed through structured interviews by three licensed pediatric-psychiatric clinicians according to the DSM-5 criteria (American Psychiatric Association, 2013). Information about type and timing of exposure to maltreatment was collected by child welfare facility staffs who knew the background of a participating child well. The number of children who had experienced physical abuse, emotional abuse, sexual abuse, and neglect was seven (33.3%), 11 (52.4%), two (9.5%), and 16 (76.2%), respectively, when multiple responses for maltreatment types were allowed. The mean duration of exposure was 7.7 ± 4.68 years. The Mini-International Neuropsychiatric Interview for Children and Adolescents was administered to exclude other psychiatric conditions (e.g., PTSD) (Sheehan et al., 2010). All children had experienced physical and/or emotional abuse and/or neglect early in life prior to coming into care. The Trauma Symptom Checklist for Children (TSCC) (Briere, 1996), a 54-item self-report measure, was used to evaluate post-traumatic and other relevant symptoms found in some traumatized children (anger, anxiety, depression, post-traumatic stress, dissociation, and sexual concerns). Twenty-two TD children with no history of maltreatment were recruited as control participants from local schools or communities via advertisements, matched for age, sex, handedness, and race/ethnicity (mean age = 12.95 years; 12 girls). It was also confirmed that the TD children had no disorders meeting the DSM-IV criteria for any major Axis I and Axis II disorders (American Psychiatric Association, 1994). Participants who had a full-scale intelligence quotient (FSIQ) <70 on the Wechsler Intelligence Scale for Children-Fourth Edition (WISC-IV) or the Wechsler Adult Intelligence Scale-Third Edition (WAIS-III) (Wechsler, 1997; Wechsler, 2003) were excluded. The study protocol was approved by the Ethics Committees of the University of Fukui and was conducted in accordance with the Declaration of Helsinki. All children and a parent or director of child welfare facilities provided written informed assent and consent for participation in this study.

### 2.2 Brain imaging and analysis

Image acquisition was performed using a 3-Tesla scanner (Discovery MR 750; General Electric Medical Systems, Milwaukee, WI) with a 32-channnel head coil. A T1-weighted anatomical dataset was obtained from each subject by fast spoiled gradient recalled imaging sequence (voxel size 1 × 1 × 1 mm, echo time = 1.996 ms, repetition time = 6.38 ms, inversion time = 600 ms, flip angle = 11°, total scan time = 4 min 50 s).

Voxel-based morphometry was performed using Statistical Parametric Mapping version 12 (SPM12) (Wellcome Department of Imaging Neuroscience, University College London, London, UK; http://www.fil.ion.ucl.ac.uk/spm/software/spm12/) implemented in MATLAB 9.5 (Math-Works Inc., Natick, MA). The T1-weighted images were coarsely segmented into GM, white matter (WM), cerebrospinal fluid, and skull/scalp compartments using tissue probability maps. The Diffeomorphic Anatomical Registration through Exponentiated Lie Algebra (DARTEL) algorithm was applied to the segmented brain tissues to generate a study-specific template and to achieve an accurate inter-subject registration with improved realignment of smaller inner structures. The segmented GM images were spatially normalized into the Montréal Neurological Institute (MNI) space and were written out with an isotropic voxel resolution of 1.5 mm. Any volume change induced by normalization was adjusted via a modulation algorithm. Spatially normalized GM images were smoothed by a Gaussian kernel of 8 mm full width at half maximum.

Regional differences in GM volume between groups were analyzed in SPM 12 using two-sample *t*-test models. Potential confounding effects of age, sex, Full Scale Intelligence Quotient, and total brain volume (calculated as the sum of GM and WM volumes) were modeled and variances attributable to them were excluded from the analyses. A whole-brain analysis with correction for multiple comparisons at the cluster level was conducted to examine regional differences in GM volume between the groups. The statistical threshold was set at *p* < 0.001 at the voxel level and *p* < 0.05 with family-wise error correction for multiple comparisons at the cluster level. The anatomical localization of significant clusters was investigated with the Automated Anatomical Labeling and Brodmann area (BA) atlases implemented in the MRIcron software package (Rorden et al., 2007).

### 2.3 Random forest regression with conditional trees

The presence of a potential ‘sensitive period,’ during which exposure to childhood maltreatment may be more strongly associated with regional alterations in GM volume, was assessed using random forest regression with conditional inference trees (‘cforest’in R package party; (Strobl et al., 2007)), as we have used in previous studies (Pechtel et al., 2014; Schalinski and Teicher, 2015; Teicher et al., 2018; Tomoda et al., 2012). This is a form of machine learning in which a large number of unpruned decision trees are generated and their results aggregated. Random forest regression has the advantage of high accuracy, no restrictions regarding the distribution and scaling properties of the data, high tolerance for multicollinearity, does not assume a linear relationship between degree of exposure and outcome, can detect and model interactive effects between predictor variables, and provides a novel means of determining variable importance (Breiman, 2001; Cutler et al., 2007). We used a variant of Breiman's approach with conditional trees as the base learners to avoid a potential problem with biased estimates, which can emerge when variables differ in range or number of categories (Strobl et al., 2007). Conditional forest regression indicates importance by assessing the decrease in accuracy, as noted by the increase in mean square error (MSE), of the forest's fit following permutation of a given predictor variable. Permutation of important predictor variables produces a large increase in MSE, whereas permutation of unimportant predictors produces little or no increase in MSE.

While random forest regression is well-suited to identify ages when severity of exposure has the most important predictive effect on outcome (Choi et al., 2012; Tomoda et al., 2012), we also sought to determine whether the magnitude of importance at peak periods could have occurred by chance. Hence, we used a re-randomization test in which we calculated the maximal increase in MSE with severity of exposure at any age in the original dataset, and then tested for this degree of increase in MSE in 10,000 alternative random forests regressions in which the association between regional volume and exposure histories was randomly reshuffled.

Two separate analyses were conducted to evaluate the importance of potential predictors of regional alterations in GM volume. In the first analysis, we determined the importance of exposure during specific time periods including in utero exposure to mother experiencing domestic violence and direct exposure to neglect or physical, emotional, or sexual abuse from birth to 16 years of age, annually. Years were scored either zero or one, for exposure to any type of maltreatment during the year. Duration of abuse was included as an additional predictor to control for the potential confounding relationship between early exposure and longer duration.

In the second analysis random forest regression was used to determine the comparative importance of exposure to neglect versus physical, emotional, or sexual abuse. The number of types of maltreatment was included as an additional predictor because multiplicity of exposure may be a more important determinant than any specific type of exposure. For these analyses we used the β scores of regional signal, which was centered and scaled to provide an arbitrary mean of 100 and SD of 10. Each forest consisted of 200 trees with four variables randomly selected for evaluation at each node.

## 3 Results

### 3.1 Structural MRI data

**Fig. 1:**
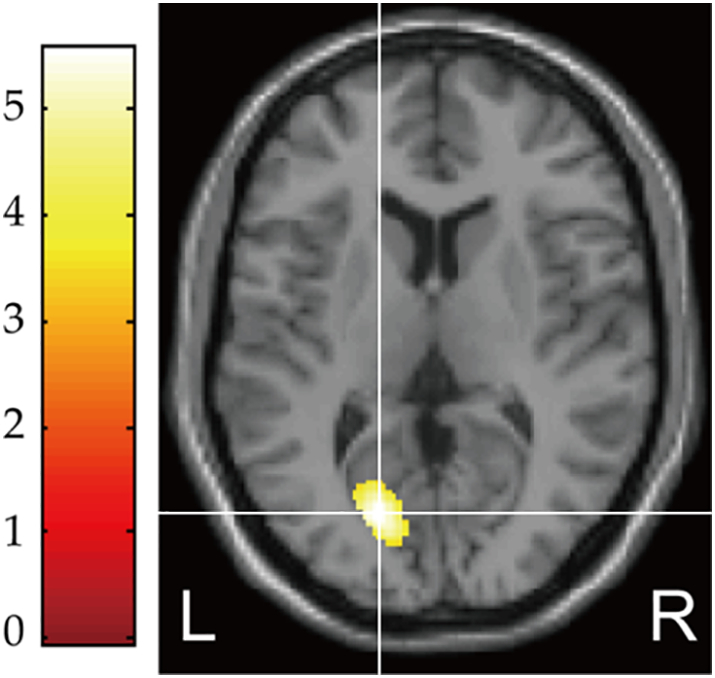
Structural differences in regional gray matter (GM) volume between the typically developing (TD) and reactive attachment disorder (RAD) groups. The RAD group showed significantly reduced GM volume in the left primary visual cortex (BA 17) compared to the TD group (*p* = 0.038, FWE-corrected cluster level). Color scales represent t-values.

As shown in Fig. 1, in line with our previous research (Shimada et al., 2015), the GM volume was significantly reduced by 20.6% in the left primary visual cortex of the RAD group compared to the TD group (BA 17; MNI coordinates, *x* = −20, *y* = −74, *z* = 8; cluster size = 644 voxels, *p* = 0.038, family-wise error-corrected cluster level). The GM eigenvariates (i.e., linearly transformed estimates of GM volume) from the identified cluster were extracted and used for regression analyses to confirm the effects of type and timing of maltreatment for the GM volume reduction.

### 3.2 Age of exposure

**Fig. 2:**
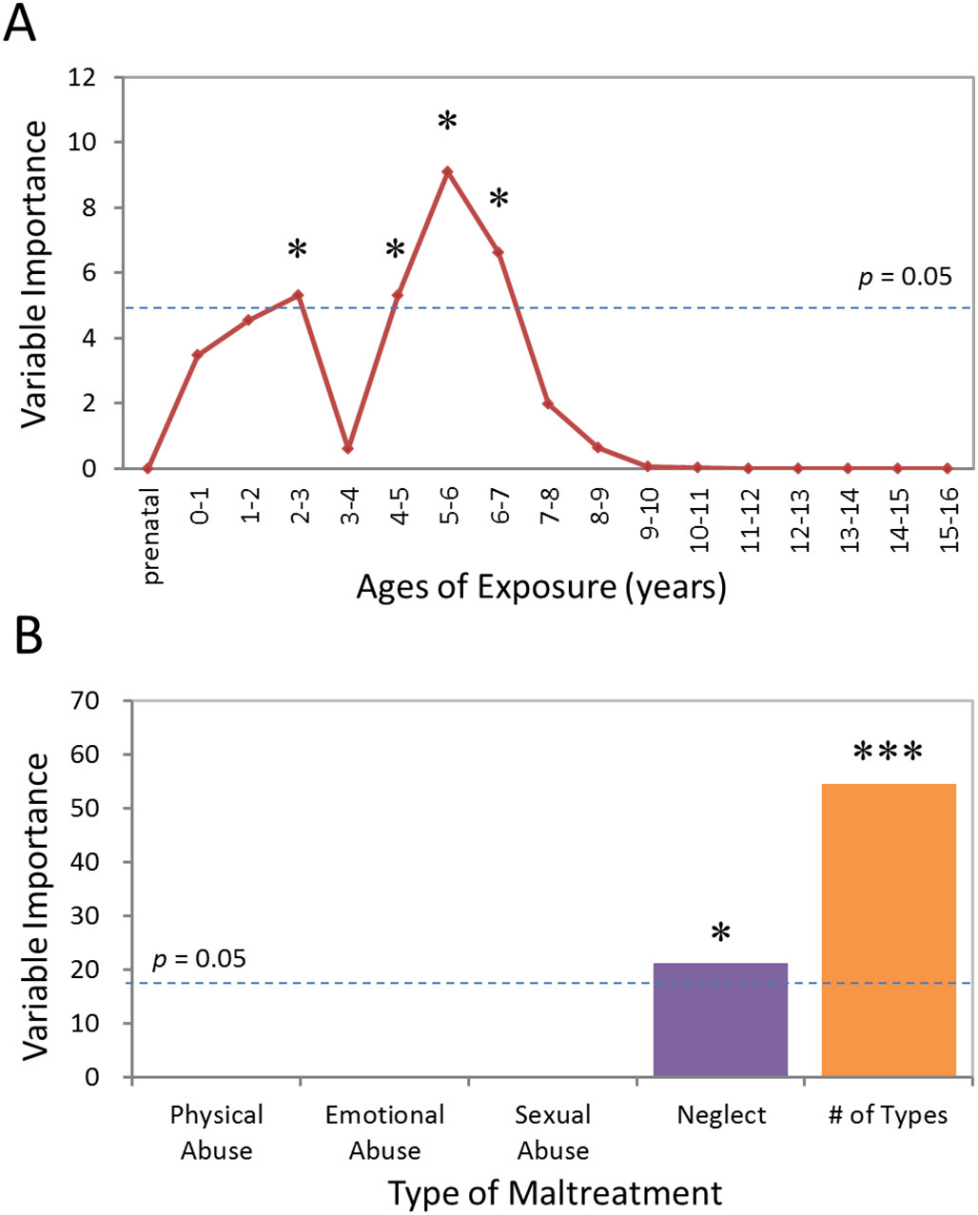
(A) Maximal sensitivity by age of exposure (maximal importance of age of exposure, regardless of type) in RAD. Results of a random forest regression with conditional trees indicated the importance of exposure to early maltreatment from birth to 16 years of age on the GM eigenvariates for the left visual cortex. Importance is indicated by degradation in fit, as indicated by the increase in mean square error (MSE), following effective elimination of each age from the model by permutation. (B) Maximal sensitivity by type and number of maltreatments (maximal importance of type and number of maltreatments, regardless of age) in RAD. * *p* < 0.05; *** *p* < 0.001 (FDR-corrected for multiple comparisons).

As seen in Fig. 2A, the most important temporal predictor of the GM eigenvariates in the left primary visual cortex of the RAD group was whether children were exposed to maltreatment at 4–7 years of age, with a peak occurring at 5–6 years (*p* < 0.05, false-discovery rate (FDR) corrected for each peak of age, respectively). The probability of obtaining these three dots (4–5, 5–6, and 6–7 years) of age with this combined degree of importance was significantly low (*p* < 0.005). The next most important age was 2–3 years (*p* < 0.05, FDR corrected). Altogether, importance was high from 2 to 7 years of age with the exception of 3–4 years. Predicted response based on only these temporal factors correlated (*r* = 0.493) with actual response (*p* < 0.05).

### 3.3 Type of maltreatment

As shown in Fig. 2B, the degree of the GM eigenvariates in the left visual cortex of the RAD group could also be predicted with reasonable accuracy based on type of maltreatment and number of types of maltreatment (*r* = 0.650, *p* < 0.05). Number of types of maltreatment was the most important predictor (*p* < 0.05, FDR corrected). Neglect as a specific type of maltreatment emerged as the second most important predictor (*p* < 0.05, FDR corrected, respectively), and the likelihood of obtaining two measures with this degree of importance was significantly low (*p* < 0.001).

### 3.4 Relationships between sensitivity and clinical symptom measures

**Fig. 3:**
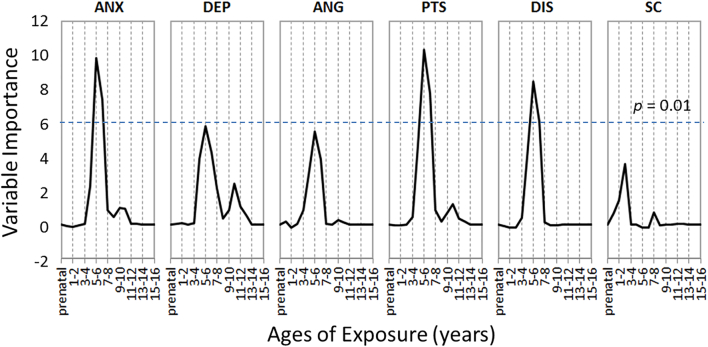
Sensitive periods of maltreatment exposure for each symptom of RAD. ANX: Anxiety, DEP: Depression, ANG: Anger, PTS: Post-traumatic stress, DIS: Dissociation, SC: Sexual concern. A dotted line represents a 0.01 *p*-value threshold FDR-corrected for multiple comparisons.

We also performed random forest regression analyses to further characterize the relationship between sensitivity for type and timing of maltreatment and various psychiatric symptoms with RAD. Subscale scores of the TSCC were considered as the evaluation scale for each symptom caused by child maltreatment and used as a dependent variable in regression analyses. As indicated in Fig. 3, the analysis showed that the significant temporal predictor for psychiatric symptom measures of anxiety, PTSD, and dissociation problems in the RAD group was, consistent with the sensitive period to the GM volume reduction, whether children were exposed to maltreatment at 4–7 years of age (*p* < 0.01, FDR corrected for each peak of age, respectively). This result suggests that these symptoms in maltreated children may be attributed to a decrease in GM volume. In contrast, the analyses did not reveal any significant associations between the type of maltreatment and psychiatric symptoms (*p* > 0.01, FDR corrected for each type, respectively).

## 4 Discussion

In the present study, we investigated the effect of type and timing of maltreatment on structural alteration of the visual cortex in children and adolescents with RAD. Our findings revealed that there is a potential sensitive period between 5 and 7 years of age for GM volume reduction in the left primary visual cortex (BA17) as a result of maltreatment. In addition, we also confirmed that symptoms such as anxiety, PTSD, and dissociation showed the same sensitive period as RAD. We also found that number of types of maltreatment and neglect as a specific type of maltreatment had significant effect on the GM volume reduction. The present study provides the first evidence showing that type and timing of maltreatment have important roles in inducing structural abnormalities and symptoms in children and adolescents with RAD.

The present findings revealed a potential sensitive period, between 5 and 7 years of age, when maltreatment exerted maximal effects on GM volume reduction in the visual cortex in children and adolescents with RAD. This is in line with our earlier observation that childhood sexual abuse was associated with a reduction in occipital cortex GM volume if it occurred prior to age 12 years (in an all-female sample) but not after (Tomoda et al., 2009a). Why the sensitive period for visual cortex abnormality in RAD was between 5 and 7 years is an interesting question. In general, it is well known that brain development in this period is most significant, as the capacity of the brain reaches approximately 90% of adult volume by age 6 years (Stiles and Jernigan, 2010). Similarly, a classical animal study showed that plasticity of the visual cortex abates following puberty (Hubel and Wiesel, 1998). Normal development of human visual perception has also suggested that some aspects of visual function, such as letter acuity, global motion, and perhaps light sensitivity in the midperiphery, end at 6 or 7 years of age (Lewis and Maurer, 2005). Therefore, adverse experiences in this period may tend to have an effect on brain disorganization and dysfunction in the primary visual cortex.

The present study is relatively unique in its focus on the potential consequences of exposure to a specific type of abuse. This approach has been useful in revealing similarities and differences between the neurobiological correlates of exposure to childhood sexual abuse (Andersen et al., 2008; Tomoda et al., 2009a), parental verbal abuse (Choi et al., 2009; Tomoda et al., 2011), witnessing domestic violence (Choi et al., 2012; Tomoda et al., 2012), and harsh corporal punishment (Sheu et al., 2010; Tomoda et al., 2009b). We found that the number of types of maltreatment was the most important predictor of brain alteration. This finding is largely consistent with the findings of earlier studies that found that the number of types of maltreatment is positively related to GM volume reduction or altered functional connectivity, although the deficit areas were partly inconsistent (Jedd et al., 2015; Takiguchi et al., 2015; Teicher et al., 2012). Meanwhile, numerous studies have shown that the number of adverse childhood experiences has negative effects on mental health (e.g., (Edwards et al., 2003; Teicher et al., 2006)). Therefore, multiplicity of the type of maltreatment is involved in the degree of structural and/or functional alterations and may be reflected in the severity of the symptoms of RAD and result in poorer mental health condition.

We also found that neglect as a specific type of maltreatment emerged as the second most important predictor. Although shutdown dissociation is one of the major symptoms of RAD, a previous study reported that emotional neglect was associated with the severity of shutdown dissociation (Schalinski and Teicher, 2015). Similarly, shutdown dissociation strength in patients with PTSD is associated with the response of the visual cortex to emotional visual stimuli (Schalinski et al., 2014). Hence, neglect may have an important role in the association between visual cortex alterations and the atypical response of the visual cortex, and the symptom of shutdown dissociation may depend on structural alteration of the visual cortex. On the other hand, given that neglect as a type of maltreatment was not as strong a predictor as the number of types of maltreatment, it can be assumed that exposure to neglect was too common among the children with RAD (76.2% as described in the Method) to have sufficient variance. For this reason, neglect may play a role as a basis for the adverse effect of the multiplicity of maltreatment rather than having direct effects.

In addition, it is noteworthy that there is not one sensitive period but different sensitive periods during which visual input is necessary for the normal development of different aspects of vision (Blakemore, 1988; Daw et al., 1992; Harwerth et al., 1986; Jones et al., 1984; Singer, 1988). While we found similarities in GM volume reduction in the visual cortex in individuals who had witnessed domestic violence in our prior observation, differences were also revealed in the sensitive periods between 11 and 13 years (Tomoda et al., 2012). Although the exact neural mechanisms underlying the different sensitive period for the organization of similar brain areas (visual cortex) remain unknown, environmental inputs for vision based on maltreatment types (neglect or witnessing domestic violence) may each have different sensitive periods and their interaction may have a crucial role in the disorganization of the visual cortex in children and adolescents with RAD.

Further analysis also revealed the same temporal sensitive period, between 5 and 7 years of age, for symptoms such as anxiety, PTSD, and dissociation. The visual cortex has been found as part of a neurocircuit that regulates the stress response to emotional visual images (Teicher and Samson, 2013). Although the primary visual cortex and limbic system, including the amygdala and hippocampus, are anatomically connected through the inferior longitudinal fasciculus (ILF) (Amaral et al., 2003; Catani et al., 2003), the ILF as a visual-limbic pathway subserves emotional functions specific to the visual modality. Thus, the primary visual cortex conveys emotional signals (e.g., fearful images) to the amygdala and receives feedback signals from the amygdala based on this anatomical connection (Adolphs, 2004; Vuilleumier, 2005). A functional connectivity analysis using MRI has revealed that the effect of the amygdala on behavior to emotional visual stimuli was mediated through backward projections from the amygdala to the visual cortex (Lim et al., 2009; Pessoa and Adolphs, 2010). In addition, exposure to early life stress may be involved in the atypical development of the visual-limbic pathway. In a recent structural MRI study using a diffusion tensor imaging technique, a reduction in white matter tract integrity of the ILF was observed in adults who witnessed domestic violence and were exposed to maltreatment during childhood (Choi et al., 2012; Huang et al., 2012). Hence, alterations in the development of the visual cortex with dysfunction of the limbic system may play a significant role in the occurrence of RAD symptoms with involvement of limbic system dysfunction, including anxiety, PTSD, and dissociation.

Some limitations of the present study should be noted. First, the main limitation is the relatively small patient group as also described in our prior study. Although our adopted technique using machine learning to obtain the statistics is relatively robust for the sample size, further research involving a larger number of subjects is desirable to generalize our results. Second, the present study used a retrospective design to identify the type and timing of maltreatment that precludes the identification of causal links between RAD and the GM volume differences. Prospective longitudinal studies are required to establish a causal relationship between GM volume decrease and maltreatment. Finally, there is a potential limitation in the diagnosis of RAD. As previously suggested (Kay and Green, 2013; Minnis et al., 2013; Zeanah and Gleason, 2015), a child may be given only a ‘suspected’ diagnosis of RAD because the diagnosis is not definitive. A more robust diagnosis needs to be based on ecological observation of the child's interaction activity in a local school or community, as well as on clinical interview and questionnaire data (Minnis et al., 2013). Nevertheless, this study sheds light on the significance of type and timing of environmental input to develop neural dysfunction consistent with RAD.

## 5 Conclusion

In conclusion, we provided novel evidence that type and timing of maltreatment have a significant role in inducing structural abnormalities in the left occipital visual cortex in children and adolescents with RAD. These findings are consistent with those of previous studies with adults who had experienced childhood maltreatment; adverse experience in this early period may affect the development of the primary visual system, reflected in GM volume reduction in the visual cortex. These visual cortex GM volume abnormalities may also be associated with the symptoms of RAD or increased risk for later psychopathology. As suggested in the current study, the symptoms of RAD are expected to vary in impact based on the type, timing, and severity of maltreatment exposure coupled with a number of susceptibility and resilience cofactors. We recently hypothesized and have approached using the term “ecophenotype” to delineate how these factors differentially affect psychiatric disorders (Teicher and Samson, 2013; Teicher et al., 2016). This approach will lead to a better understanding of differences in the clinical presentation of RAD. Understanding of the neural basis underpinning these differences may be useful for establishing a more accurate clinical assessment and treatment monitoring and enable some individuals to more effectively compensate for the abnormalities.
